# Single-session clearance of complex impacted choledocholithiasis using a novel large-channel cholangioscope and a 4.5-Fr electrohydraulic lithotripsy probe

**DOI:** 10.1055/a-2971-5445

**Published:** 2026-10-28

**Authors:** Ankoor H Patel, Michael Ma, Petros Benias, Arvind J Trindade

**Affiliations:** 1 Medicine, Division of Gastroenterology 12287 Rutgers University School of MedicineNew BrunswickNew JerseyUnited States; 2 RWJ ﻿Barnabas HealthNew BrunswickNew JerseyUnited States

A 59-year-old woman was admitted with right upper quadrant abdominal pain and jaundice. A computed tomographic scan showed a dilated bile duct with a large burden of large complex choledocholithiasis. Endoscopic retrograde cholangiopancreatography (ERCP) was performed with the cholangiogram confirming a dilated duct with impacted large stones throughout the bile duct ( Fig. 1 ). The impacted nature of the stones ( Figs. 1 and 2 ) did not allow for basket capturing of the stone for mechanical lithotripsy.

**Fig. 1:**
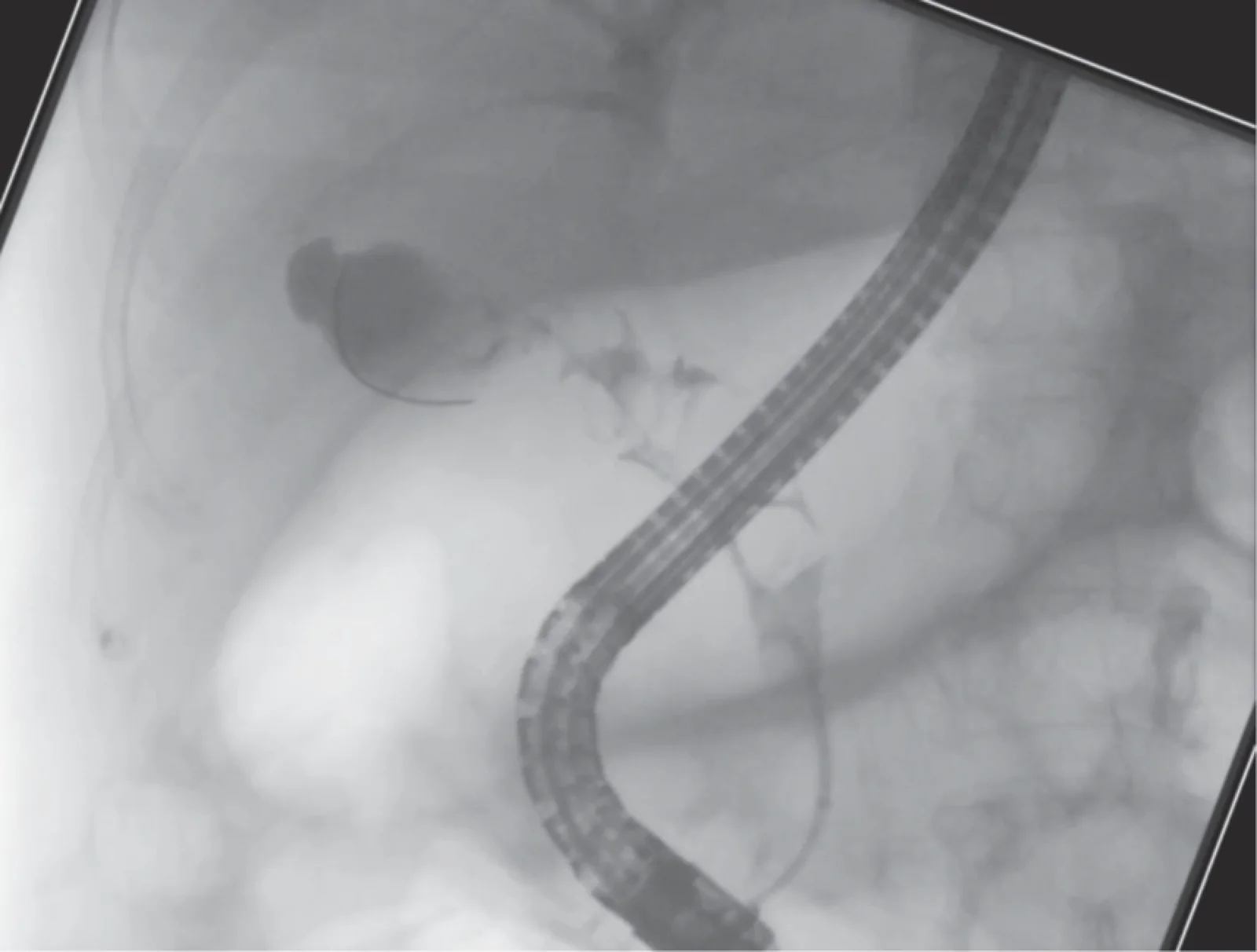
A cholangiogram showing large complex choledocholithiasis.

**Fig. 2:**
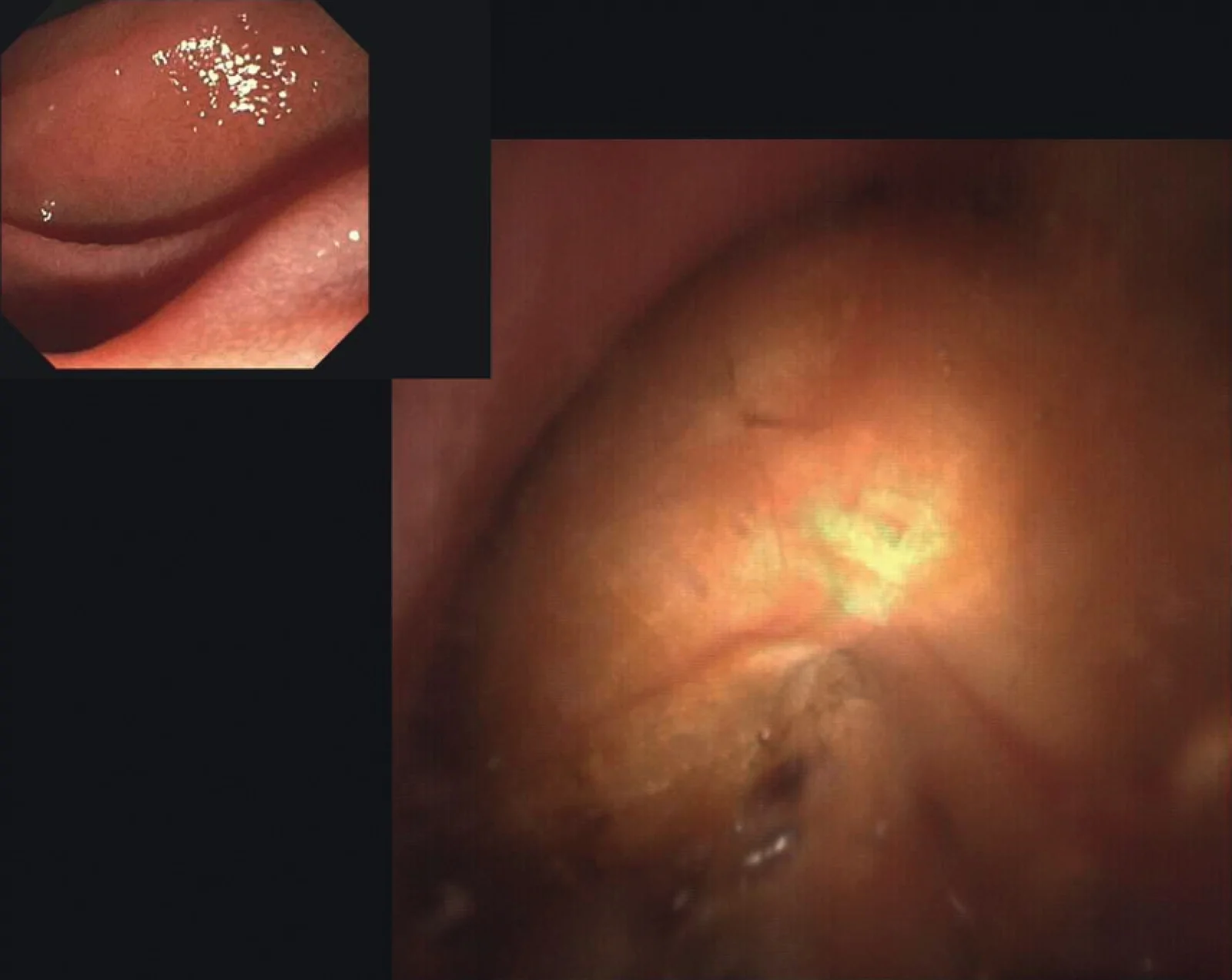
A cholangioscopic image of a large stone occupying the entire bile duct lumen with the electrohydraulic lithotripsy probe cracking the stone.

The decision was made to use a novel cholangioscope with a large diameter 1.7 mm channel to allow for a large 4.5Fr electrohydraulic lithotripsy (EHL) probe (Dragonfly Pancreaticobiliary System, Medtronic, MN, USA). EHL was performed using a novel system (Waltz Lithotron EL27 Compact EHL System, Rohrdorf, Germany), and to our surprise efficiently broke up the stones in under 5 minutes ( Video 1 ) using setting C (high intensity, 950 mJ, at a maximum of 40 Hz). The stones were then completely removed using a stone extraction balloon all in one session. In our experience, the use of the 4.5Fr probe allowed for a powerful and efficient stone destruction. Most importantly, it allowed for a single session ERCP. In our experience, this stone burden would typically require multiple ERCPs for ultimate stone clearance using older EHL systems with a 1.9Fr EHL probe.

**Video 1** A video showing cholangioscopy using a large 4.5Fr electrohydraulic lithotripsy probe for the management of complex large stone burden. Video link below:


In conclusion, the use of novel cholangioscopes with a large diameter channel allows for the efficient destruction of large stones with a 4.5 Fr EHL probe, with the potential of avoiding follow up ERCPs. There are two available cholangioscopes that allow for the usage of a large caliber EHL probe in the United States. A summary of the technical aspects of currently available cholangioscopes in the United States is summarized in Table 1 .

**Table:** **Table 1** Comparison of contemporary digital cholangioscopy platforms

Characteristics	Dragonfly	SpyGlass DS II	eyeMAX 1.2	eyeMAX 2.0
Outer diameter, mm	3.5	3.5	3.2	3.9
Working length, mm	1760	2140	2200	2200
Working channel diameter, mm	1.7	1.2	1.2	2.0
Up/down angulation	90°	90°	90°	90°
Right/left angulation	Full shaft rotation	90°	90°	90°
Shaft flexibility ^a^	Highly flexible	Improved maneuverability	Improved maneuverability	Improved maneuverability
Image resolution (K)	160	62.5	160	160
Biopsy forceps opening width, mm	6.0	4.1	3.0	4.5
Biopsy cup/jaw width, mm	1.5	1.0	1.0	1.6
Rotational biopsy forceps compatibility	Yes	No	No	No
Maximum lithotripsy probe compatibility, Fr	4.5	3 ^b^	3.0	4.5

Abbreviation: Fr, French size.

Note: Objective technical specifications are shown where available; qualitative characteristics are reported according to manufacturer specifications.

Technical specifications of currently available digital single-operator cholangioscopy systems. Specifications are based on manufacturer-reported data.

^a^ Manufacturer-reported qualitative specification.

^b^ Commercially supplied lithotripsy probe is 1.9 Fr despite a 3-Fr compatible channel.

Endoscopy_UCTN_Code_TTT_1AR_2AL

## Declaration of GenAI Use

The authors confirm that no generative artificial intelligence was used in the preparation of this manuscript.

